# Focused ultrasound-mediated enhancement of blood–brain barrier permeability for brain tumor treatment: a systematic review of clinical trials

**DOI:** 10.1007/s11060-024-04795-z

**Published:** 2024-08-29

**Authors:** Honglin Zhu, Caitlin Allwin, Monica G. Bassous, Antonios N. Pouliopoulos

**Affiliations:** 1 Faculty of Life Sciences and Medicine, King’s College London, London, UK; 2 School of Biomedical Engineering & Imaging Sciences, King’s College London, London, UK; 3 School of Cancer and Pharmaceutical Sciences, King’s College London, London, UK

**Keywords:** Focused ultrasound, Blood-brain barrier, Microbubbles, Glioblastoma, Brain tumor

## Abstract

**Purpose:**

Brain tumors, particularly glioblastoma multiforme (GBM), present significant prognostic challenges despite multimodal therapies, including surgical resection, chemotherapy, and radiotherapy. One major obstacle is the limited drug delivery across the blood–brain barrier (BBB). Focused ultrasound (FUS) combined with systemically administered microbubbles has emerged as a non-invasive, targeted, and reversible approach to transiently open the BBB, thus enhancing drug delivery. This review examines clinical trials employing BBB opening techniques to optimise pharmacotherapy for brain tumors, evaluates current challenges, and proposes directions for further research.

**Methods:**

A systematic literature search was conducted in PubMed and ClinicalTrials.gov up to November 2023, searching for “ultrasound” AND “brain tumor”. The search yielded 1446 results. After screening by title and abstract, followed by full-text screening (n = 48), 35 studies were included in the analysis.

**Results:**

Our analysis includes data from 11 published studies and 24 ongoing trials. The predominant focus of these studies is on glioma, including GMB and astrocytoma. One paper investigated brain metastasis from breast cancer. Evidence indicates that FUS facilitates BBB opening and enhances drug uptake following sonication. Exploration of FUS in the pediatric population is limited, with no published studies and only three ongoing trials dedicated to this demographic.

**Conclusion:**

FUS is a promising strategy to safely disrupt the BBB, enabling precise and non-invasive lesion targeting, and enhance drug delivery. However, pharmacokinetic studies are required to quantitatively assess improvements in drug uptake. Most studies are phase I clinical trials, and long-term follow-up investigating patient outcomes is essential to evaluate the clinical benefit of this treatment approach. Further studies involving diverse populations and pathologies will be beneficial.

## Introduction

Brain tumors continue to exhibit a poor prognosis, with less than 20% of patients surviving beyond 5 years post-diagnosis [1]. Glioblastoma multiforme (GBM) represents the most prevalent primary malignant brain tumor. Despite various therapeutic modalities, including surgery, chemotherapy and radiotherapy, substantial improvements in patient survival have not been realised [2]. A major contributing factor is the challenge of pharmacotherapies penetrating the blood–brain barrier (BBB) to reach the tumor at therapeutic concentrations [3]. Consequently, there is growing interest in research focused on safe and reversible BBB opening (BBBO), which holds promise for enhancing the delivery and efficacy of therapeutic agents [4].

The BBB, formed by microvascular endothelial cells that regulate molecule and ion transfer from the blood into the brain parenchyma, enables homeostasis and normal neuronal functioning [3]. These cells interact with astrocytes and pericytes to uphold the barrier’s integrity [5]. The challenge of drug delivery is compounded by localized vascular changes in tumors, which can increase interstitial fluid pressure (IFP), complicating the dynamics of drug delivery [6].

Several techniques are used for transient BBBO. One involves administering hyperosmotic agents like mannitol via intra-arterial infusion [7]. However, the dilutional effects of collateral arterial system in the Circle of Willis complicates its reproducibility [8]. Another method is convection-enhanced delivery (CED), which relies on the principles of bulk flow and uses stereotactic catheter to administer therapeutics directly into the target. However, backflow presents as a significant challenge, where the infusion penetrates through the catheter tract rather than reaching the targeted area [9]. This results in a dilutional effect at the tumor site, as the drug therapy advances to unintended areas. Other techniques, such as implanting drug-releasing polymers, and conjugation of pharmacotherapies to proteins, also face drawbacks (e.g. reduced delivery and rapid clearance from circulation) [10].

**Fig. 1:**
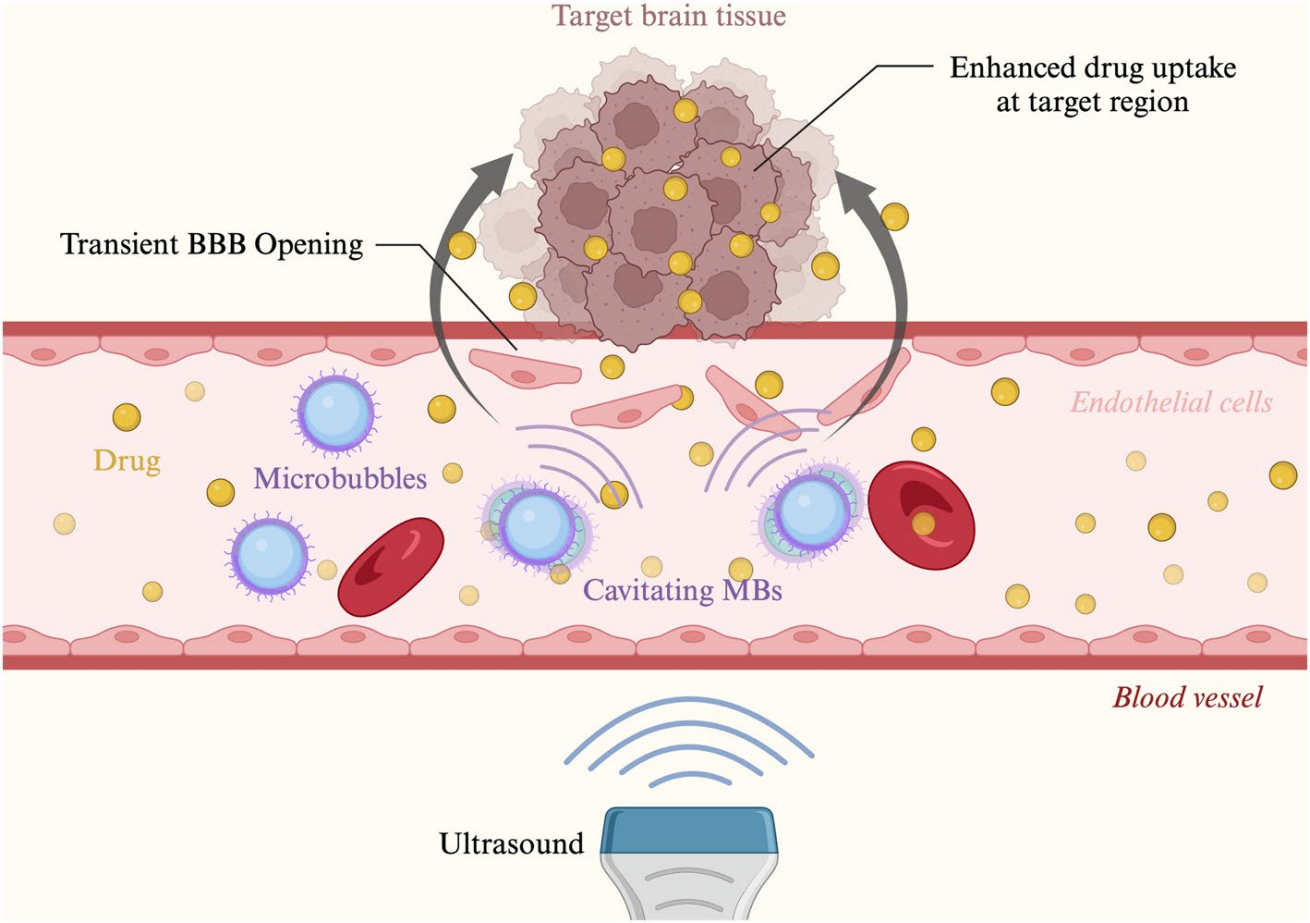
Schematic of FUS-Induced BBB Disruption. The application of ultrasound waves to targeted brain region induces microbubble-seeded acoustic cavitation. This process generates intravascular shear stress, leading to reversible changes in BBB permeability, thereby facilitating the enhanced penetration of therapeutic agents into brain tissue. Adapted from “Lipid-Based Microbubbles (MBs) as Ultrasound-Based Drug Delivery System” by BioRender.com (2024). Retrieved from https://app.biorender.com/biorender-templates

The use of focused ultrasound (FUS) to transiently open the BBB is under increasing research. This technique involves directing low-frequency ultrasound waves at targeted brain regions, producing microbubble-seeded acoustic cavitation and intravascular shear stress that can produce reversible permeability changes in the BBB (Fig. 1). The disruption allows therapeutic agents, such as chemotherapeutics or gene therapies, to penetrate brain tissue more effectively. FUS-mediated BBBO is non-invasive and can be precisely controlled, making it a promising approach for treating brain tumors and other neurological disorders while minimizing systemic side effects [11]. Currently, the use of ultrasound with microbubbles is the only non-invasive, targeted, and reversible method for transient BBBO to enhance drug delivery [12].

Microbubbles are micron-sized, gas-filled particles that are widely used as contrast agents in diagnostic ultrasound imaging. When coupled with therapeutic ultrasound, they can enhance targeted drug delivery by locally amplifying intravascular stresses. Low-frequency, low-intensity FUS causes microbubbles to oscillate in response to the alternating phases of the acoustic waves, causing temporary BBB disruption [6, 12]. Drugs can be administered either concurrently with microbubbles or bound to their shell via ligands for localized release [13].

Pre-clinical studies on mice, rats, rabbits, canines, and non-human primates (NHPs) have demonstrated safe, effective, and reversible BBBO with ultrasound and microbubbles, leading to human clinical trials to assess efficacy and safety in clinical settings [14–19]. For example, evidence has shown that FUS-mediated BBBO is safe in mice with diffuse intrinsic pontine gliomas (DIPG) [20–22]. Other studies have shown that BBBO does not affect cognitive performance of NHPs post-treatment, further indicating the safety of FUS [17, 23].

This review investigates current clinical trials on FUS-guided BBBO during pharmacotherapy administration for brain tumors, offering a comprehensive analysis of the current clinical landscape of FUS use in neuro-oncology. We critically assess existing challenges surrounding this treatment method and propose directions for further research.

## Methods

The systematic literature search was conducted in PubMed and ClinicalTrials.gov from inception to 1st November 2023, searching for a combination of “ultrasound” AND “brain tumor”. The exact search terms used were [(focused ultrasound) OR (unfocused ultrasound) OR (pulsed ultrasound) OR (microbubble*)) AND ((glioma) OR (glioblastoma) OR (astrocytoma) OR (ependymoma) OR (medulloblastoma) OR (brain tumour*) OR (brain tumor*) OR (brain neoplasm*)].

**Fig. 2:**
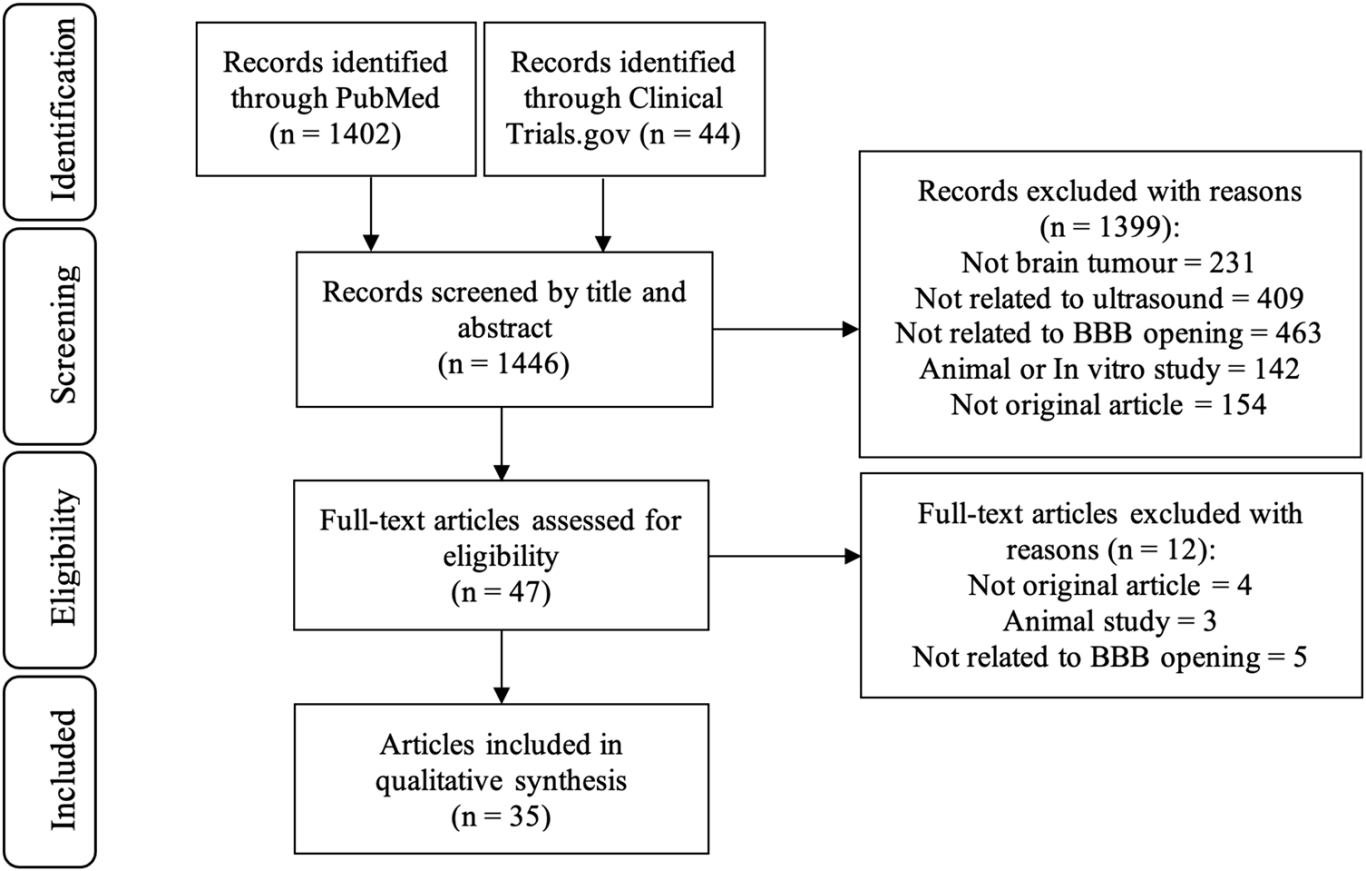
PRISMA flow chart illustrating the identification and selection process

Our literature search yielded 1446 results (Fig. 2). Two investigators (HZ and CA) independently determined eligibility of study after screening by title and abstract. Discrepancies were discussed and resolved through discussion with a therapeutic ultrasound (ANP) or pharmacy (MGB) expert. Studies were then screened by full text (n = 47), adhering to inclusion and exclusion criteria:

### Inclusion criteria

Included studies were published in English involving participants with brain tumors, investigated the use of ultrasound to open the BBB, and reported relevant outcomes as primary or secondary endpoints.

### Exclusion criteria

Excluded studies were not relevant to brain tumor treatment, lacked ultrasound intervention, or used FUS for thermal ablation or sonobiopsy but not BBBO for drug delivery. Additionally, we excluded animal and in vitro studies, as well as non-original work, e.g. reviews, comments, editorials, letters, and opinion articles.

## Results

### Published studies

After screening, data was collected from 11 publications across 6 centers involving 7 clinical trials and 61 patients in total. The majority of studies were Phase 0 or Phase I trials evaluating the safety and feasibility of BBBO through FUS treatment (Table 1).

**Table 1:** Details of published clinical trials on FUS-induced BBB opening for brain tumor treatment

Study	Sonabend et al. [25]	Meng et al. [24]	Park et al. [29] and 2021 [30]	Anastasiadis et al. [32]	Chen et al. [33, 34]	Carpentier et al. [26]; Idbaih et al. [27]; Asquier et al. [28]	Mainprize et al. [31]
NCT number	NCT04528680	NCT03714243	NCT03712293	NCT03322813	NCT03626896	NCT02253212	NCT02343991
Publication overview	Provides first direct evidence that LIPU-MB substantially increases the brain concentration of systemically administered drugs in human	First-in-human report of non-invasitve, spatially targeted monoclonal antibody delivery across the BBB with MRgFUS	This study opened a new therapeutic strategy for GBM by combining BBB disruption with a larger molecular agent for six cycles of standard temozolomide (TMZ) chemotherapy period for the first time	Demonstrate the capability of this technology to tune BBB opening in real-time, creating opportunities for improved treatment of brain tumor	Provides preliminary evidence of FUS-induced immune modulation as an additional therapeutic benefit by converting the immunosuppressive TME into an immunostimulatory TME via a higher but safe FUS dosage	A first-in-man, single-arm, single- center trial to investigate the transient disruption of the BBB in patients with recurrent GBM	First report of targeted chemotherapy delivery using MRgFUS—to evaluate BBB disruption in humans quantitatively—quantification of the penetration of TMZ via MRgFUS
Centre	Northwestern Memorial Hospital, Chicago	Sunnybrook Research Institute, Toronto	Yonsei University College of Medicine, Seoul	University of Maryland School of Medicine, Baltimore	Chang Gung Memorial Hospital at Linkou, Taiwan	Assistance Publique–Hopitaux de Paris (AP-HP) University Hospital La Pitie- Salpetriere, Paris	Sunnybrook Research Institute, Toronto
Trial stage (at time of publication)	Phase I	N/A	N/A	Phase 0	Phase I	Phase I/IIa	Phase I
Number of patients, n	17, 9 M 8 F	4, 4 F	6, 2 F 4 M	4, 1 F 3 M	6, 3 F 3 M	19, 6 F 13 M	5, 1 F 4 M
Age, mean (range)	57 (33–72)	45.25 (31–56)	55.8 (50–67)	32.5 (29–36)	49.5 ± 19.4 (32–80)	59 (38–77)	55.8 (33–71)
Condition	Recurrent glioblastoma (IDH wild-type)	HER-2 positive breast cancer brain metastasis, ER/PR positive	Grade IV glioblastoma, IDH wild, MGMT methylation 4 +ve 2 −ve, 1p19q codeletion negative, EGFR negative	Intrinsic glioma, IDH unmutated; Diffuse infiltrating glioma (II) Oligodendroglioma (II) Anaplastic oligodendroglioma (III)	Recurrent GBM	Recurrent GBM; IDH 1 wild-type 17/19; IDH2 wild-type 18/19	Glioma—Grade IV astrocytoma (n = 3)—Grade III anaplastic astrocytoma (n = 2)
Primary outcome	Evaluate safety and maximal tolerated dose of albumin-bound paclitaxel after LIPU-MB based BBBO	Evaluate safety and treatment related AEs through clinical neurological exams and neuroimaging studies	Evaluate safety and treatment related AEs through clinical neurological exams and neuroimaging studies	Assess safety and feasibility of M–BFUS for BBBO in non-enhancing regions of infiltrating glioma, and related AEs	Evaluate safety of transient BBB opening by the NaviFUS System in recurrent GBM: DLT, AEs, physical and neurological examination, KPS, mini-mental state examination (MMSE), vital signs, and clinical laboratory tests	Evaluate safety and tolerance to sonication with the SonoCloud-1 device and to determine the MTD of ultrasound	Safety through clinical neurologic exam and radiologic evidence of haemorrhage, swelling or mass effect; technical feasibility determined by contrast enhancement in target regions with resolution within the 24 h
MRI pre and post BBBO	Y (1–2 d pre; 1 h post)	Y (pre, 1 h and 18–24 h post)	Y (2 d pre, STAT post)	Y (pre, post)	Y (pre, 24 h post)	Y (2 d pre, 30 min post)	Y (pre, 24 h post)
MRI findings post BBBO	Diminished 1 h after sonification	Hypointense spots 7/20 (35%) resolved over time	one pseudoprogression	2/4 (50%) show new GRE/T2 changes within targeted region	FUS induced BBB opening resolves within 24 h	BBB disruption detected in 30 min post-FUS MRI	Contrast extravasation in grid pattern that resolves within around 20 h
Chemo drug and dose	Albumin-bound paclitaxel (Six dose levels at 40 mg/m^2^, 80 mg/m^2^, 135 mg/m^2^, 175 mg/m^2^, 215 mg/m^2^, 260 mg/m^2^) ± Carboplatin	Trastuzumab	Temozolomide (TMZ) 1st cycle—150 mg/m^2^, 2nd–th cycle—200 mg/m^2^	Fluorecein	N/A	IV Carboplatin	IV Liposomal doxorubicin (n = 1) PO Temozolomide (TMZ) (n = 4)
Frequency	Every 3 weeks for 3 cycles (2–6)	Up to 6 cycles	Up to 6 cycles	N/A	Once	2.1 cycle on average (2–4 cycles)	Once
Ultrasound system	SonoCloud-9 [SC9]; CarThera, Lyon, France; implanted device	ExAblate 4000-system Type 2 (INSIGHTEC)	ExAblate Neuro Model 4000 Type 2.0 220 kHz system, InSightec, Haifa, Israel	ExAblate 4000-system Type 2	NaviFUS	SonoCloud-1, CarThera	ExAblate Neuro (InSightec Tirat Carmel, Israel) system
FUS parameters	N/A	13 ± 6 W; 27 ± 7 cm^3^ (sonication volume)	6.28 ± 2.19 W	Average 3.38–24.55 W, Max 47.27 W	500 kHz; 0.48, 0.58, 0.68 MI	0.41, 0.53, 0.66, 0.78, 0.90, 1.03, 1.15 Mpa; pulse repetition frequency of either 0.5 or 1 MHz (1.2 or 2.4% duty cycle)	220 kHz, 4–15 W, each sonication was delivered at 0.74% duty cycle for 50 s
FUS duration	4 min 30 s	N/A	79.89 s	N/A	120 s	150–270 s	50 s per cycle
Microbubbles	Perflutren lipid microsphere Definity 10 μL/kg; Lantheus, North Billerica, MA, USA	Microbubble ultrasound contrast agent (DEFINITY, Lantheus)	Definity [perflutren lipid micro- sphere], Lantheus Medical Imaging, Inc	DEFINITY microbubbles	SonoVue, 0.1 ml/kg; maximal 4.8 ml	SonoVue, 0.1 ml/kg; maximal 4.8 ml	Definity ® (4 μl/kg)
Effect on uptake	Increase in mean parenchymal paclitaxel concentration from 0.037 μM [95% CI 0.022–0.063] in nonsonicated to 0.139 μM [0.083–0.232] in sonicated [3.7-time increase], and carboplatin (from 0.991 μM [0.562–1·747] in nonsonicated to 5.878 μM [3.462–9.980] μM in sonicated [5.9-times increase])	450% increase in uptake under voxel-based analysis	N/A	N/A	CE-T1 and Ktrans maps showed statistically significant SIC when comparing the time points at immediate (0.5 h) and 24 h after FUS, from 9.32 ± 12.47 to 5.25 ± 1.67% in CE-T1; and 0.0113 ± 0.0031 to 0.0018 ± 0.0011 min^−1^ in Ktrans	52/65 sonification showed evidence of BBBD on MRI; The degree of BBBD increased with acoustic pressure as 0% (0.41 MPa), 0% (0.53 MPa), 18% (0.66 MPa), 57% (0.78 MPa), 80% (0.90 MPa), 77% (1.03 MPa), and 66% (1.15 MPa)	Average contrast enhancement of 35%, Chemotherapy concentration enhancement of 47% (increased from 0.15 to 0.22 (ng/mg) post-sonication), and 671% (0.45 × 10^−4^ to 3.47 × 10^−4^ post-sonication) in two patients, respectively
Inclusion/exclusion criteria	≥ 18 yr; tumor diameter ≤ 70 mm; Karnofsky performance status ≥ 70	18–80 yr; metastatic Her2-positive breast cancer with brain metastases; Karnofsky performance status ≥ 70	18–80 yr; Grade IV GBM; Karnofsky performance status ≥ 70; previous gross total surgical resection	21–85 yr; suspected infiltrating glioma; Karnofsky performance status ≥ 70	≥ 20 yr; recurrent GBM; Karnofsky performance status > 60	Recurrent GBM; after at least a first-line standard of care; tumor diameter < 35 mm	18–80 yr; evidence of malignant glioma; Karnofsky performance status ≥ 70
Adverse effects	Encephalopathy (2/12, 16.7%); Peripheral neuropathy (1/12, 8.33%); Neutropenia (8/17, 47%); leukopenia (5/17, 29%); hypertension (5/17, 29%); seizure (3/17, 17.65%); transient headaches (12/17, 71%); paraesthesia (2/17, 12%), facial/limb weakness (4/17, 24%), dysphasia (2/17, 12%), dysarthria (2/17, 12%), dysaesthesia (3/17, 18%); blurred vision (5/17, 29%)	Pin-site tenderness (1/20, 5%), Back discomfort (1/20, 5%), Headache (1/20, 5%)	Mild motor weakness relieved after steroid (1/6); pseudoprogression at site of BBBD (1/6); 1 with grade 2 hematological adverse event that was related to TMZ (1/6)	None	None determined to be related to FUS treatment or MB	Neurological deficits (2/19) disppeared after 15 d; fatigue (23%); haematological disorders (32%); headache (26%); brain edema (11%); faintness (11%)	Minor headache at the helmet attachment sites (n = 2)
Follow up, months	11.89 (IQR 11.12–12.78); 10/17 (59%) died due to diseaes progression; 100% had disease progression; median progression free survival was 2.9 m (95% CI 2.7–4·.6) and overall survival was 11 m (7·95–not reached)	Follow up MRI scheduled at 1 m and 3 m post treatment	12.17 ± 1.94 months, 2 with recurrence at 8 and 2 m. 1 underwent surgery, 1 restarted with TMZ. 100% survival rate up to 1 year, and the other four patients are on observation without recurrence	15.7 m (mean), no tumor recurrence	37 days after NaviFUS treament/30 days after definitive resection surgery	1 yr follow up; Patients with no/poor BBB disruption (n = 8) had a median progression-free survival (PFS) of 2.73 m, and median overall survival (OS) of 8.64 m. Patients with clear BBBD (n = 11) had a median PFS of 4.11 m, and median OS of 12.94 m	1 week, 1 months, 3 months

Glioma, particularly recurrent GBM, is most frequently investigated for FUS treatment. Other types of gliomas investigated include astrocytoma, oligodendroglioma, and diffuse infiltrating glioma. One study investigated brain metastasis from human epidermal growth factor receptor 2 (HER-2) positive breast cancer [24]. Notably, all studies focused on adult patients, with a mean age of 50.7 years, and none of the published studies has thus far explored the pediatric population.

The drugs used following FUS-guided BBBO included paclitaxel [25] and carboplatin [26–28] for recurrent GBM, and temozolomide (TMZ) for GBM [29, 30] and astrocytoma [31]. Trastuzumab was used for HER-2 positive breast cancer brain metastasis [24].

Trials utilized three ultrasound systems, including the SonoCloud (CarThera, France), ExAblate Neuro (InSightec, Israel), and NaviFUS (Taiwan). SonoCloud system is an ultrasound device implanted during craniotomy for tumor removal. This allows for repeated BBBO over multiple chemotherapy cycles. ExAblate Neuro is an MR-guided hemispherical multi-element array, which has electronic steering capabilities and high targeting precision. The NaviFUS system is neuronavigation-guided and can be used in an outpatient setting outside the MRI.

Few studies reported evidence suggestive of enhanced drug uptake post-sonication. In one study the mean parenchymal paclitaxel concentration increased by 3.7-fold (from 0.037 to 0.139 μM) in treated patients, and carboplatin by 5.9-fold (from 0.991 to 5.878 μM) [25]. Another study showed a 35% average contrast enhancement, and chemotherapy concentration enhancements of 47 and 671% post-sonication in two patients, respectively [31].

MRI demonstrated FUS-induced BBBO, evident from discrete contrast extravasation on gadolinium-enhanced MRI immediately post-treatment [24, 25, 31]. The contrast extravasation occurred in a grid pattern with ExAblate which resolved within 24 h [24, 31], and in a cylindrical pattern with SonoCloud-9, which resolved within an hour [25]. Immediate side effects included transient headache, pin-site tenderness, and neurological deficits associated with sonicated regions, including weakness, dysarthria, and dysphasia. Side effects generally diminish with steroid treatment [29], and resolved within 1 to 48 h in one study [27]. In a phase I trial, patients receiving 260 mg/m^2^ of albumin-bound paclitaxel experienced grade 2 and 3 encephalopathy with low-intensity pulsed ultrasound and concomitant administration of intravenous microbubbles (LIPU-MB) [25]. This dose-limiting toxicity resolved, and treatment was recommenced at lower doses of 175 and 215 mg/m^2^, respectively. Additionally, neutropenia, leukopenia, and hypertension commonly manifested as grade 3–4 treatment-emergent adverse events [25].

Long-term patient outcomes in phase 0/I trials are often limited due to short follow-up periods. Those studies predominantly focus on safety and feasibility, as well as determining maximum safe dosage of drugs. Consequently, many studies have yet to report long-term outcomes, with follow-ups ranging from 1 to 15 months, and some omitting results entirely.

### Ongoing trials

There are 24 ongoing trials currently investigating the use of FUS in neuro-oncology (Table 2). Similar to the published trials, the majority are in their early phases, with only 2 in phase III. 12 ongoing trials utilised the ExAblate device, 6 used SonoCloud, and 6 used neuronavigation-guided transducers (NaviFUS and UltraNav systems).

**Table 2:** Details of current ongoing clinical trials, including medications and drug properties

NCT number	Trial	Centre	Conditions	Device	Medication	Medication class	Molecular weight	Lipophilicity (Partition coefficient: n-octanol–water [Pow])	Phase
NCT05879120	Randomized Study of Neo-adjuvant and Adjuvant Pembrolizumab with and Without Targeted Blood Brain	MD Anderson Cancer Center, Houston, Texas, US	Grade IV glioma (glioblastoma or	ExAblate	Pembrolizumab	Monoclonal IgG4 kappa anti-PD1 antibody	149,000 Da	N/A^a^	II
NCT04021420	Safety and Efficacy of Sonocloud Device Combined with Nivolumab in Brain Metastases from Patients With Melanoma	Saint-Louis Hospital, Paris, France	Patients with histologically	SonoCloud	Nivolumab	Anti-PD1 monoclonal antibody	143,597.4 Da	N/A^a^	I + II
NCT05762419	FUS Etoposide for DMG—A Feasibility Study	Columbia University Irving Medical Center, New York, US	Diffuse Intrinsic Pontine Glioma	UltraNav	Etoposide	Topoisomerase II inhibitor	588.6 Da	0.45	I
NCT05630209	Blood Brain Barrier (BBB) Disruption Using Exablate Focused Ultrasound with Doxorubicin for Treatment of	Children's National Medical Center, Washington, US; Nicklaus Children's Hospital Miami, Florida, US	Brain Tumor	ExAblate	Doxorubicin	Anthracyline	543.5 Da	0.82	I + II
NCT04804709	Non-Invasive Focused Ultrasound (FUS) With Oral Panobinostat in Children with Progressive Diffuse Midline	Columbia University Irving Medical Center/NewYork-Presbyterian Hospital New York, US	Diffuse Intrinsic Pontine Glioma	UltraNav	Panobinostat	Pan-deacetylase inhibitor	349.4 Da	3.56	I
NCT03744026	Safety and Efficacy of Transient Opening of the Blood–brain Barrier (BBB) With the SonoCloud-9	Northwestern Memorial Hospital, Chicago (same centre as NCT04528680)	Glioblastoma, Adult	SonoCloud-9	Carboplatin	Second-generation platinum compound	371.3 Da	N/A^b^	I/IIa
NCT04446416	Efficacy and Safety of NaviFUS System add-on Bevacizumab (BEV) in Recurrent GBM Patients	Linkou Chang Gung Memorial Hospital, Taoyuan City, Taiwan	Glioblastoma Multiforme	NaviFUS System	Bevacizumab	Anti-VEGF monoclonal IgG1 antibody	149,000 Da	N/A^a^	N/A
NCT04667715	Safety and Effectiveness of Blood–Brain Barrier Disruption (BBBD) in Subjects with Suspected Infiltrating Glioma	University of Maryland, Baltimore, Maryland, US, The University of Texas MD Anderson Cancer Center, Houston,	Glioma	ExAblate	N/A	N/A	N/A	N/A	N/A
NCT03551249	Assessment of Safety and Feasibility of ExAblate Blood–Brain Barrier (BBB) Disruption	University of Maryland, Baltimore, Maryland, US; Brigham and Women's Hospital, Boston, Massachusetts, US, University	Glioblastoma	ExAblate	Temozolomide	DNA alkylating agent	194.2 Da	1.07	N/A
NCT05293197	Safety Study of the Repeated Opening of the Blood–brain Barrier with the SonoCloud Device to Treat Malignant Brain	Institut Curie, Paris, France, Service de neurochirugie Pédiatrique—Hôpital Necker—Enfants Malades, Paris, France	Primary Malignant Brain	SonoCloud	Carboplatin	Second-generation platinum compound	371.3 Da	N/A^b^	I
NCT04417088	Exablate Blood–Brain Barrier Disruption for the Treatment of rGBM in Subjects Undergoing Carboplatin Monotherapy	Stanford University, Palo Alto, California, US; University of Maryland, Baltimore, Maryland, US; Brigham and Women's	Recurrent Glioblastoma	ExAblate	Carboplatin	Second-generation platinum compound	371.3 Da	N/A^b^	I + II
NCT02253212	Safety of BBB Opening with the SonoCloud	Groupe Hospitalier Pitié Salpetriere—Neurosurgery Department, Paris, France	Glioblastoma	SonoCloud	Carboplatin	Second-generation platinum compound	371.3 Da	N/A^b^	I + I
NCT04063514	The Use of Focused Ultrasound and DCE K-trans Imaging to Evaluate Permeability of the Blood–Brain Barrier	Neurological Associates of West LA, Santa Monica, California, US	Low Grade Glioma of Brain	Brainsonix	N/A	N/A	N/A	N/A	N/A
NCT04998864	Assessment of Safety and Feasibility of ExAblate Blood–Brain Barrier (BBB) Disruption in GBM Patients	Fondazione IRCCS Neurologico Carlo Besta, Milano, Italy, CINAC-Hospital HM Puerta del Sur Móstoles, Madrid, Spain	Glioblastoma	ExAblate	Temozolomide	DNA alkylating agent	194.2 Da	1.07	N/A
NCT05383872	Blood–Brain Barrier Disruption (BBBD) for Liquid Biopsy in Subjects with GlioBlastoma Brain Tumors	University of California, Los Angeles, California, US	Glioblastoma, Glioma	ExAblate	N/A	N/A	N/A	N/A	N/A
NCT03616860	Assessment of Safety and Feasibility of ExAblate Blood–Brain Barrier (BBB) Disruption for Treatment of Glioma	Sunnybrook Health Sciences Centre, Toronto, Ontario, Canada	Glioblastoma	ExAblate	Temozolomide	DNA alkylating agent	194.2 Da	1.07	N/A
NCT05317858	Blood–brain Barrier (BBB) Disruption Using Exablate Focused Ultrasound with Standard of Care Treatment of	St. Joseph's Hospital and Medical Center, Phoenix, Arizona, United States, Miami Cancer Institute at Baptist Health,	Brain Metastases of Non-small Cell	ExAblate	Pembrolizumab	Monoclonal IgG4 kappa anti-PD1 antibody	149,000 Da	N/A^a^	III
NCT03626896	Safety of BBB Disruption Using NaviFUS System in Recurrent Glioblastoma Multiforme (GBM) Patients	Linkou Chang Gung Memorial Hospital, Taoyuan City, Taiwan	Recurrent Glioblastoma	NaviFUS System	N/A	N/A	N/A	N/A	N/A
NCT04528680	Ultrasound-based Blood–brain Barrier Opening and Albumin-bound Paclitaxel and Carboplatin for Recurrent Glioblastoma	Northwestern Memorial Hospital, Chicago, Illinois, US	Glioblastoma	SonoCloud	Paclitaxel, Carboplatin	Microtubule stabiliser; Second-generation platinum compound	853.9 Da; 371.3 Da	4.35; N/A^b^	I + II
NCT05615623	Blood Brain Barrier (BBB) Disruption Using Exablate Focused Ultrasound with Doxorubicin for Treatment of	Sunnybrook Research Institute, Toronto, Ontario, Canada	Brain tumor	ExAblate	Doxorubicin	Anthracyline	543.5 Da	0.82	I + II
NCT05902169	Sonocloud-9 in Association with Carboplatin Versus Standard-of-Care Chemotherapies (CCNU or TMZ) in	Northwestern University, Chicago, Illinois, US; NewYork-Presbyterian/Columbia University Irving Medical Center,	Glioblastoma	SonoCloud	Carboplatin, Lomustine, Temozolomide	Second-generation platinum compound; Alkylating agent; DNA	371.3 Da; 233.7 Da; 194.2 Da	N/A^b^; 2.90; 1.07	III
NCT04440358	Exablate Blood–Brain Barrier Disruption with Carboplatin for the Treatment of rGBM	Sunnybrook Health Sciences Centre Toronto, Ontario, Canada; Fondazione IRCCS Neurologico Carlo Besta, Milano,	Recurrent Glioblastoma	ExAblate	Carboplatin	Second-generation platinum compound	371.3 Da	N/A^b^	I + II
NCT04988750	Evaluate the Safety and Preliminary Efficacy of the Combination of NaviFUS System with Re-irradiation for	University of Virginia, Charlottesville, Virginia, US	Glioblastoma	NaviFUS System	ALA	Photosensitising drug	131.1 Da	− 0.40	I
NCT05733312	Extracellular Impact of Ultrasound-induced Blood–brain Barrier Disruption	Mayo Clinic Minnesota, Rochester, Minnesota, US	Brain Tumor	ExAblate	N/A	N/A	N/A	N/A	N/A

The molecular weight of drugs was sourced from https://pubchem.ncbi.nlm.nih.gov/

^a^Pembrolizumab, Nivolumab, and Bevacizumab are monoclonal antibodies with no meaningful Pow due to large size and hydrophilic nature

^b^Carboplatin is a platinum-based drug, and its Pow is not provided in standard resources

A variety of drugs, including carboplatin, doxorubicin, bevacizumab, are investigated. Each of these drugs has distinct molecular properties, such as molecular weight or lipophilicity, enabling them to readily pass through the BBB with the assistance of FUS-mediated BBBO, as shown in pre-clinical trials [35–37]. For example, the molecular mass of carboplatin is 371 Da, whereas bevacizumab has a mass of 149 kDa (Table 2). This variation in molecular mass results in different drug delivery enhancement when using FUS, even with identical treatment parameters.

Additionally, there are differences in the treatment pathway. Most studies focus on enhanced drug delivery following BBBO. Another pathway is to use FUS to open the BBB to mark the regions of infiltrating gliomas, in order to improve visualization during surgical resection and maximize total tumor resection. Currently, one ongoing trial (NCT04667715) is evaluating this endpoint, pointing the direction for further research.

## Discussion

### Pharmacotherapies

A range of medications in combination with FUS treatments are under investigation. TMZ, the first-line therapy of high-grade gliomas, exhibits high oral bioavailability and the ability to cross the BBB due to its lipophilicity and small size. Despite its efficacy, its cerebrospinal fluid concentration is only about 20% of plasma concentration [38], and the median survival in GBM patients following traditional treatment with surgery, radiation, and TMZ is only 14.6 months [38]. This limitation may stem from efflux by the P-glycoprotein 1 (P-gp), a common multidrug resistant protein abundant in BBB within cancerous tissues, as shown in rats [39]. Nonetheless, P-gp is shown to be down-regulated after treatment with FUS and microbubbles [40], and TMZ concentration has been shown to increase by 7.7-fold when BBBO is performed concomitantly [29]. The promising result following FUS, coupled with its inherent potency, renders it an ideal candidate for FUS trials. Notably, two published trials have already examined the effect of this drug with FUS [31, 34].

Albumin-bound paclitaxel is another medication that showed promising effects after treatment with LIPU-MB. As a chemotherapeutic agent, paclitaxel is 1400 times more potent than TMZ [25]. However, despite its potency, paclitaxel does not cross the BBB [41], and has not shown efficacy for glioma in clinical trials [42].

### Devices

Magnetic resonance-guided focused ultrasound (MRgFUS) offers a promising approach, as multiple studies have demonstrated its ability to temporarily disrupt the BBB without damaging surrounding tissues [43]. MRgFUS delivers ultrasound energy with intraoperative imaging guidance and real-time feedback, enabling non-invasive, selective targeting of intracranial lesions, including those in deep and functionally critical regions [30].

Implant-based approaches for BBBO, such as the implantable SonoCloud device by CarThera, are beneficial as they can be implanted immediately following surgical removal of tumor, thereby avoiding the need for additional procedures. However, they are constrained by the direction of the transducer and have limited ability to precisely control the direction of BBBO (Table 3). In contrast, MRgFUS offers greater flexibility in selecting the target location and size, as the direction of ultrasound can be adjusted. Their ease of use and lack of need for targeting in each session make these transducers attractive for regular treatments in the same region.

**Table 3:** Summary of different types of ultrasound devices used in neuro-oncology, their features, and distinct advantages and disadvantages

Ultrasound device	Summary	Targeting scheme	No. of transducers	Advantages	Disadvantages	Trial
SonoCloud® (CarThera Inc.)	Implanted ultrasound device for repeated BBB opening	N/A	9	No skull distortion; No need for treatment planning; Low cost	Requires surgery for implantation; Limited ability to precisely control direction of sonication or treatment location	NCT04528680; NCT02253212
ExAblate® (InSightec Inc.)	Extracorporeal fixed stereotactic frame-based MRI-guided device	Electronic focusing; MRI guidance	1024	Non-invasive; High precision; Electronic steering; BBB opening uniformity	Requires head stabilization; Requires MRI so patients need to be stable and not claustrophobic; High cost	NCT03626896
NaviFUS® (NaviFUS Inc.)	Frameless neuronavigation-guided device	Electronic focusing; Neuronavigation guidance	256	Non-invasive; Outpatient; Does not require in-line MRI guidance	Low registration precision for neuronavigation; Potential head movement	NCT05123534; NCT03714243; NCT03712293; NCT02343991
UltraNav® (Delsona Therapeutics)	Frameless neuronavigation-guided device	Geometric Focusing; Neuronavigation guidance	1	Non-invasive; Outpatient; Does not require in-line MRI guidance	Low registration precision for neuronavigation; Potential head movement; No electronic steering/fixed focus; No aberration correction	NCT05762419

MRgFUS disrupts the BBB through multiple mechanisms, such as direct disruption of tight junctions and induced transcytosis [44]. Intraoperative MRI enables the identification of bioeffects caused by BBB disruption, potentially reducing the risk of false-negative outcomes compared to implant-based methods. MRgFUS can also target any brain region with minimal tissue reflection at the tissue-skull boundary, especially when the stereotaxic frame is appropriately positioned. Furthermore, real-time acoustic feedback and power modulation facilitate precise control and adaptation of the BBBO magnitude and distribution, enhancing safety and efficacy [30].

However, MRgFUS procedures require fixation of stereotaxic frame with regular frame adjustments, which may cause discomfort and emotional stress [30]. The time and cost of MRI also needs to be considered. Additionally, as enhanced T1-weighted MRI is the gold standard for BBBO confirmation, gadolinium contrast needs to be administered, and as such, patients with poor renal function are often excluded.

Other methods that monitor microbubble activity such as passive acoustic detection and acoustic mapping could be used for predicting the outcome of FUS treatments and degree of drug delivery enhancement. However, these techniques have their own limitations, such as variable sensitivity, limited resolution, and computational speeds [45–47]. Imaging microbubble acoustic emissions in 2D and 3D can identify the spatial location of microbubble activity, which can be correlated with the degree of gadolinium penetration into the brain, a typical surrogate for BBBO confirmation, or directly with the degree of drug delivery [48]. All devices incorporate cavitation monitoring as a feedback mechanism, apart from CarThera.

### Trial variability

The number of participants in ongoing trials is often limited, ranging from 3 to 57. The recently initiated SONOBIRD study, with around 560 participants enrolled across the globe (NCT05902169), will provide invaluable information on treatment response in a large cohort. Small sample sizes have limited the generalizability of trials, affecting the establishment of formalized standards for evaluating drug choice, device type, and treatment parameters. Variations exist in acoustic pressure/intensity, pulse length, center frequency, pulse repetition frequency, and total treatment time. These differences make it challenging to interpret the effects of ultrasound parameters, especially given the limited data on drug uptake. There appears to be a positive correlation between the number of cycles and duration of treatment with a higher incidence of side effects in some studies [28, 37]. More comparative studies are needed to evaluate the exact correlation due to the limited data available. Other parameters such as ethnicity, comorbidities, age, and grade of tumor, all interplay into the prognosis and suitability of each treatment.

Variability also exists in microbubble parameters among studies. The two microbubbles used across published trials are Definity (perflutren lipid microspheres, 4 or 10 µL/kg) and SonoVue (sulfur hexafluoride, 0.1 mL/kg, max 4.8 mL). A study in rats suggest similar BBBO effects under equivalent concentrations [49]. Future research should aim to standardize microbubble usage and dosing protocols to better monitor concentration effects in patients.

### Safety

Appropriate ultrasound parameters are crucial to avoid risks such as erythrocyte extravasations in cerebral microvasculature, limiting the incremental ultrasound level below 0.8 mechanical index (MI) [50]. MRI abnormalities following FUS treatment include T2* hyperintensities within 24 h post-treatment, indicating brain edema, and susceptibility-weighted imaging hypointensities, indicating localized microhemorrhage [24].

### Potential improvements to clinical trials

Only a few studies have reported quantitative data regarding change in drug concentrations post-FUS [25, 31]. Moreover, information on the restoration rate of BBB integrity is not generally provided, with limited exceptions, showing restoration within a few hours after procedure with SonoCloud [25]. Additionally, parameters such as pulse length, intensity, and pulse repetition frequency should be standardized to enable better comparisons of outcomes across different studies.

Furthermore, additional investigation is needed to assess the feasibility and specific considerations for treatment across diverse populations. No published studies have evaluated treatment feasibility in pediatrics, though there are ongoing trials for diffuse midline glioma (DMG) patients. DMG, also known as DIPG, is a rare brain tumor that primarily occurs in children between 2 and 9 years of age, with a poor prognosis and an average survival of 9–12 months after diagnosis [51]. DMG is well protected from circulating drugs due to intact BBB. Additionally, surgical resection is in general not feasible, due to its location within the brainstem and neighboring eloquent areas. These characteristics render FUS a promising therapeutic solution for DMG. Currently, there are three ongoing trials using FUS to enhance the delivery of etoposide, panobinostat, and doxorubicin for DMG, respectively, with additional studies in the planning stages. Moreover, pediatric patients require careful assessment due to anatomical variances and different neurodevelopmental stages. Common tumor types also differ, with medulloblastoma being the most prevalent. Trials targeting prominent pediatric tumor types are essential for advancing FUS applications.

### Future directions

Currently, most published studies are in initial stages with small sample sizes. The poor prognosis of brain tumors complicates long-term follow-up for assessing the efficacy of FUS. Many trials focus on short-term safety, with follow-up periods often less than two years, as longer follow-up times are often ambitious given the disease course of brain tumors. Long-term patient outcomes are necessary to establish the validity and efficacy of the approach, which has the potential to inform future treatment guidelines and clinical practice. Additional trial data, coupled with molecular imaging techniques, will provide more defined understanding of the relationship between FUS dose, drug pharmacokinetics, and tumor response [24].

FUS holds promise beyond brain tumor treatment, with applications in other brain pathologies. In Alzheimer's disease, FUS-mediated BBB disruption is shown to reduce beta-amyloid and tau pathology [52]. Its feasibility has also been explored in amyotrophic lateral sclerosis (ALS) [53]. FUS-mediated BBBO may open avenues for otherwise incurable conditions, and further research is required to fully explore these possibilities.

Further exploration is needed in developing new small- or large-molecule pharmacotherapies for GBM, with various trials currently ongoing. In a placebo-controlled phase III trial, cediranib, an oral pan-vascular endothelial growth factor (VEGF) receptor tyrosine kinase inhibitor, did not improve progression-free survival in patients with recurrent GBM [54]. Despite this, it may benefit from concomitant FUS-mediated BBBO to improve clinical efficacy. The same applies to other drugs like tivozanib and sunitinib [55, 56]. Furthermore, promising in vitro chemotherapeutic agents should undergo investigation with FUS [57]. Additionally, targeted immunotherapies, such as monoclonal antibodies or CAR-T cell therapies, could benefit from localized and reversible FUS-mediated BBBO in brain tumor patients [58].

## Conclusion

This systematic review summarized the published and ongoing clinical trials using FUS for targeted BBBO in brain tumors. Our findings indicate that FUS-mediated BBBO is a safe procedure with the potential to improve clinical outcomes. We also discussed challenges and areas for further study. Future research should aim to develop standardized, evidence-based protocols for drug and device choices, and treatment parameters for both adult and pediatric patients. Various device types and personalized pharmacotherapies should also be explored. Beyond the scope of brain tumors, FUS may benefit other conditions once its advantages and device accessibility are established.

## Data Availability

No datasets were generated or analysed during the current study.
